# Accurate estimation of isoelectric point of protein and peptide based on amino acid sequences

**DOI:** 10.1093/bioinformatics/btv674

**Published:** 2015-11-14

**Authors:** Enrique Audain, Yassel Ramos, Henning Hermjakob, Darren R. Flower, Yasset Perez-Riverol

**Affiliations:** ^1^ Department of Proteomics, Center of Molecular Immunology,; ^2^ Department of Proteomics, Center for Genetic Engineering and Biotechnology, Ciudad de la Habana, Cuba,; ^3^ Department European Molecular Biology Laboratory, European Bioinformatics Institute (EMBL-EBI), Wellcome Trust Genome Campus, Hinxton, Cambridge, CB10 1SD, UK and; ^4^ School of Life and Health Sciences, Aston University, Aston Triangle, Birmingham, B4 7ET, UK

## Abstract

**Motivation:** In any macromolecular polyprotic system—for example protein, DNA or RNA—the isoelectric point—commonly referred to as the p*I*—can be defined as the point of singularity in a titration curve, corresponding to the solution pH value at which the net overall surface charge—and thus the electrophoretic mobility—of the ampholyte sums to zero. Different modern analytical biochemistry and proteomics methods depend on the isoelectric point as a principal feature for protein and peptide characterization. Protein separation by isoelectric point is a critical part of 2-D gel electrophoresis, a key precursor of proteomics, where discrete spots can be digested in-gel, and proteins subsequently identified by analytical mass spectrometry. Peptide fractionation according to their p*I* is also widely used in current proteomics sample preparation procedures previous to the LC-MS/MS analysis. Therefore accurate theoretical prediction of p*I* would expedite such analysis. While such p*I* calculation is widely used, it remains largely untested, motivating our efforts to benchmark p*I* prediction methods.

**Results:** Using data from the database PIP-DB and one publically available dataset as our reference gold standard, we have undertaken the benchmarking of p*I* calculation methods. We find that methods vary in their accuracy and are highly sensitive to the choice of basis set. The machine-learning algorithms, especially the SVM-based algorithm, showed a superior performance when studying peptide mixtures. In general, learning-based p*I* prediction methods (such as Cofactor, SVM and Branca) require a large training dataset and their resulting performance will strongly depend of the quality of that data. In contrast with Iterative methods, machine-learning algorithms have the advantage of being able to add new features to improve the accuracy of prediction.

**Contact**: yperez@ebi.ac.uk

**Availability and Implementation:** The software and data are freely available at https://github.com/ypriverol/pIR.

**Supplementary information:** Supplementary data are available at *Bioinformatics* online.

## 1 Introduction

In a titration curve, the isoelectric point (p*I*) is the value at which the overall net surface charge of a macromolecular polyprotic species equals zero. Protein p*I* values are amongst the most widely determined and widely reported quantities in all of biochemistry and proteomics. The p*I* is obtained as essentially incidental information during isoelectric focusing (IEF) experiments, free flow electrophoresis (FFE), capillary electrophoresis, and in-gel electrophoresis experiments using IPG strips (Audain *et al.*, 2014; Ramos *et al.*, 2008). Electrophoresis-based separation of proteins and peptides in both free-flow and gel systems has been adapted to a wide variety of proteomics platforms in order to reduce the complexity of the studied proteome (Ramos *et al.*, 2008, 2011). In addition to the resolution and dynamic range of the fractionation technique, combining the electrophoretic separation of proteins with mass spectrometry analysis provides an orthogonal analytical method for improving protein identification in different workflows (Perez-Riverol *et al.*, 2013).

Assuming a protein to be denatured, theoretical calculation of the p*I* is typically rapid, requiring only the sequence as input (Cargile *et al.*, 2004). Most techniques exploit tabulated p*K*_a_ values for the different ionizable amino acid residues; such values are assumed to be constant regardless of structural context (Maldonado *et al.*, 2010). Many authors have reported different values for the p*K*_a_s of protein side chains and most of them are derived from measurements of side chains in isolated amino acids or from model compounds; as well as values derived from ionizable side chains *in situ* (Bjellqvist *et al.*, 1993; Lengqvist *et al.*, 2011). As many such alternative theoretical methods have been proposed, the calculation of protein p*I* values is in urgent need of benchmarking, since its accuracy remains largely untested. Extant comparison has been exiguous, using very small datasets (Patrickios and Yamasaki, 1995), peptides rather than proteins (Cargile *et al.*, 2004; Lengqvist *et al.*, 2011) or has reported poor accuracy (Henriksson *et al.*, 1995; Patrickios and Yamasaki, 1995).

We have previously described the database PIP-DB (Bunkute *et al.*, 2015), a collection of proteins, with associated experimentally determined p*I* values, as collated from the literature (Bunkute *et al.*, 2015). In this paper, we use PIP-DB as a gold standard reference for comparison, and describe the benchmarking of protein p*I* prediction. We also include a peptide dataset to evaluate the performance of p*I* prediction methods when estimating peptide p*I*s, due to the importance of properly assessing the accuracy of such prediction. As previously we combine different methods with different parameter values. Specifically, we evaluated five isoelectric point prediction algorithms: Iterative (Maldonado *et al.*, 2010; Patrickios and Yamasaki, 1995), Bjellquivst (Bjellqvist *et al.*, 1993; Cargile *et al.*, 2008), Cofactor (Cargile *et al.*, 2008), SVM (Perez-Riverol *et al.*, 2012) and Branca (Branca *et al.*, 2014); using, where appropriate, a set of alternate values for ionizable amino acid side chain p*K*_a_s.

## 2 Methods

Five different isoelectric point prediction algorithms were evaluated: Iterative (Maldonado *et al.*, 2010; Patrickios and Yamasaki, 1995), Bjellquivst (Bjellqvist *et al.*, 1993; Cargile *et al.*, 2008), Cofactor (Cargile *et al.*, 2008), SVM (Perez-Riverol *et al.*, 2012) and Branca (Branca *et al.*, 2014). The iterative model only considers the contribution of individual p*K*_a_ values to the Henderson-Hasselbach equation (Patrickios and Yamasaki, 1995). The Bjellquivst (Bjellqvist *et al.*, 1993) algorithm is based on determining the p*K*_a_ differences between closely related amino acids, and it was the first algorithm to propose a different p*K*_a_ value depending on the amino acid position in the sequence. The Cofactor algorithm (Cargile *et al.*, 2008) accounts for the effect of adjacent amino acids ±3 residues away from a charged aspartic or glutamic acid and the C-terminus, as well as applying a correction term to the corresponding p*K*_a_ values. Perez-Riverol and co-workers (Perez-Riverol *et al.*, 2012) proposed a support vector machine approach to predict the isoelectric point of peptides in electrophoretic experiments based on amino acid sequences and AAIndex properties. The Branca method (Branca *et al.*, 2014) uses p*K*_a_ value correction considering the influence of neighboring ionizable groups up to six residues away, multiplying each correction factor by the charged fraction of the neighboring ionizable group before applying it to the initial p*K*_a_ value. It also introduces the use of a statistical correction factor that depends on the number and type (Asp or Glu) of carboxylic acid side chains in the sequence.

Different p*K*_a_ values were evaluated for each method: for the iterative algorithm we include multiple p*K*_a_ sets reported previously (Supplementary Information, Table 1). The Bjellquivst method was evaluated using different p*K*_a_ correction factors for C- and N-terminus (Calibrated (Gauci *et al.*, 2008), Expasy (Gasteiger *et al.*, 2003), Skoog and default (Bjellqvist *et al.*, 1993)). The algorithms Cofactor and SVM were evaluated using the default values reported in the corresponding publications. The Branca algorithm was used with the flag *pKconstants_plain* set, and without considering additional chemical modification in the polypeptide sequence (for example, peptides derivatized with iTRAQ or TMT reagents). Detailed information of each estimation method can be found in Supplementary Information S1.

## 2.1 pIR R-package

To facilitate analysis of isoelectric point prediction for peptides and proteins, an R package (*pIR*) was developed using standard best practices for bioinformatics software development (Leprevost *et al.*, 2014; Perez-Riverol *et al.*, 2014). It provides several datasets used in the current study with the corresponding experimental and predicted isoelectric point values. It also provides a framework for reproducible analysis, allowing correlation and RMSD analysis of the predicted values; plot visualization and data processing (outlier removal, null value detection). *pIR* was implemented in R version ≥2.13.0 and is available from URL: https://github.com/ypriverol/pIR.

### 2.2 Datasets

For the protein analysis, PIP-DB (Bunkute *et al.*, 2015) (version 1.0), which contains curated protein p*I* literature data, was used to determine the accuracy of isoelectric point calculation. All proteins where sequence data was available were retrieved from PIP-DB with the corresponding experimental isoelectric point.

For the peptide analysis, a previously published dataset was used (Heller *et al.*, 2005). We utilized the PeptideProphet score to filter out low-confidence peptides from the dataset. In summary, a cellular extract of Drosophila Kc167 cells was fractionated in an isoelectric focusing Off-GEL device using 15 fractions. The tryptic proteome is separated using the isoelectric point and the experimentally derived p*I* values are reported with the final results. The identified peptides were analyzed with two different database search engines, namely PHENYX and SEQUEST, together with PeptideProphet, which is a popular post-processing peptide identification tool: a final list of 6529 peptides were used for the present study (Heller *et al.*, 2005).

In addition, we have analyzed the impact of common post-translational modifications on isoelectric point estimation using a third dataset, as published previously by Gauci *et al*. (2008). This experimental dataset was obtained using online TiO2 enrichment in combination with in-gel peptide IEF of a *Zebrafish* embryo lysate. It contains sub-populations of phosphorylated and N-terminal acetylated peptides whit the corresponding experimental p*I* values associated.

## 3 Results

### 3.1 Isoelectric point estimation of protein sequences

**Fig. 1.**
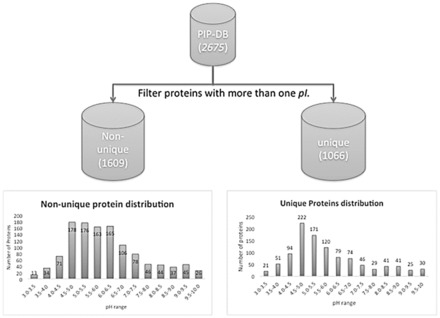
Composition of isoelectric points from PIP-DB (2675 proteins) for the two datasets: unique proteins (proteins with only one isoelectric point value, 1066 proteins); and non-unique proteins (proteins with two or more p*I* experimental values, 1609). The chart shows the protein number versus pH distribution for both subsets (pH range 3.0–10)

Initially, PIP-DB (Bunkute *et al.*, 2015) was divided into two subsets: proteins with several experimental p*I* values and proteins with a single experimental p*I* value (called unique proteins hereafter). Figure 1 shows the protein distribution by isoelectric point for both sets. The second group (proteins with only one p*I* value) contains 1066 proteins, most of which are from acid fractions (pH range 3.0–6.0).

As PIP-DB contains legacy data, not all entries were deemed useful. Initially, we isolated entries with a single measured isoelectric point rather than entries with multiple p*I* values or a range of values. Estimation of theoretical p*I* values was undertaken on the unique protein subset. Pearson correlation coefficients and root-mean square deviation (RMSD) values were used to evaluate the performance of the methods in predicting p*I.*

Table 1 summarizes the correlation coefficients and RMSD values achieved for each evaluated algorithm. The overall correlation between the experimental and theoretical p*I* values varied between *R*^2^^ ^= 0.60 (Bjellquivst—Expasy p*K*_a_ set) and *R*^2^^ ^= 0.15 (Iterative-Patrickios p*K*_a_ set). The lowest RMSD value was for the SVM algorithm (RMSD = 1.28). Most of the algorithms performed poorly when predicting protein p*I*, with a correlation coefficient between 0.55 and 0.58.

**Table 1.** Benchmark statistics for peptides and protein

	Peptide		Protein	
Method	*R* ^2^	RMSD	*R* ^2^	RMSD
SVM	0.96	0.21	0.59	1.28
ITERATIVE_GRIMSLEY	0.96	0.27	0.54	1.45
BJELL_DEFAULT	0.96	0.28	0.58	1.37
ITERATIVE_RODWELL	0.96	0.31	0.58	1.47
BJELL_CALLIBRATED	0.96	0.32	0.59	1.41
BJELL_EXPASY	0.96	0.33	0.60	1.41
ITERATIVE_THURLKILL	0.96	0.36	0.57	1.50
COFACTOR	0.86	0.44	0.57	1.39
ITERATIVE_SILLERO	0.96	0.46	0.58	1.52
ITERATIVE_TOSELAND	0.95	0.47	0.53	1.41
ITERATIVE_EMBOSS	0.96	0.48	0.57	1.54
BRANCA	0.85	0.51	–	–
BJELL_SKOOG	0.93	0.66	0.57	1.47
ITERATIVE_SOLOMON	0.93	0.71	0.57	1.48
ITERATIVE_LEHNINGER	0.93	0.71	0.57	1.48
ITERATIVE_PATRICKIOS	0.42	1.63	0.15	2.73

Pearson correlation (*R*^2^) and root-mean-square deviation (RMSD) for methods and each p*K*_a_ set. The best combination (higher *R*^2^ and low RMSD) was obtained using the support vector machine algorithm for peptides, and the Bjellquivst algorithm with the Expasy p*K*_a_ set for proteins.

**Fig. 2.**
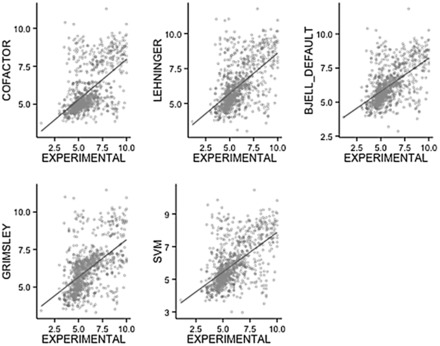
Experimental versus theoretical isoelectric point of proteins from PID-DB. Five different combinations of methods and p*K*_a_ values and algorithms were used. The *x*-axis corresponds to the experimental isoelectric point range of 3–10 and the *y*-axis is the corresponding calculated values

Figure 2 shows the correlation between the experimental value and the predicted values for five different methods. The correlation in the basic fractions (p*I* > 7.5) is inferior compared to the complete dataset. Compared to previous studies with peptides (Perez-Riverol *et al.*, 2012), the best correlation is obtained in the neutral range (5.0–7.0 pH) where fewer proteins are observed. Interestingly, the algorithms based on machine learning techniques, such as those of Cargile *et al.* (Cofactor) and Perez-Riverol *et al.* (SVM), show a similar correlation compared with the Iterative and Bjellquivst methods: 0.58 and 0.57. These results are consistent with the nature of machine learning algorithms, such as support vector machines and genetic algorithms, which depend critically on the quality and size of training datasets (Larranaga *et al.*, 2006; Perez-Riverol *et al.*, 2012).

These algorithms were developed for peptide-mixture prediction where a large number of peptides can be used to train the model (Perez-Riverol *et al.*, 2012). The recently developed Branca algorithm cannot be used to compute protein isoelectric points as it was only optimized for peptides with K or R at their C-terminus: it fails for proteins that do not have a basic C-terminus. This low correlation between experimental and predicted values demonstrated that only certain of the algorithms could be used for *in silico* studies of the isoelectric point distribution in proteomes, such as those by Wu *et al*. (2006) and Carugo (2007).

**Fig. 3.**
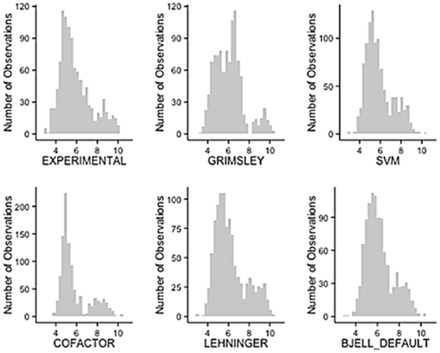
Distribution of isoelectric point for different methods and the experimental distribution. The *y*-axis is the number of proteins for the corresponding isoelectric point value (*x*-axis)

Figure 3 shows the distribution of experimental isoelectric points and predicted distributions for several algorithms. The distribution of the experimental values only shows similarity to the theoretical distribution of the SVM (Perez-Riverol *et al.*, 2012)**,** Bjellquivst (Bjellqvist *et al.*, 1993) and Iterative (with Lehninger p*K*_a_ set) algorithms. We explored the correlation between the theoretical and experimental isoelectric point of proteins for the complete PIP-DB database (Supplementary Information S2, Fig. 1) using four p*K*_a_ sets (DEFAUT, Expasy, CALIBRATED and SKOOG). The correlation for the CALIBRATED and Expasy *pK* sets is negative (*R*^2^^ ^= −0.017) for the entire PIP-DB. In contrast to peptides, over 50% of the proteins in our dataset have more than one experimental p*I* (Table 2), making it difficult to study this property properly, due to the historic use of poor analytical methods, together with protein denaturation and fragmentation (Ramos *et al.*, 2012).

**Table 2.** Protein occurrence in PIP-DB

Occurrences	1	2	3	4	5	6	7	8	9	10	11	12	13	15	>15
Number of proteins	1042	183	81	41	30	21	14	8	4	5	3	3	2	1	8

Over 50% of proteins are found with at least 2 experimental isoelectric point value associated.

**Fig. 4.**
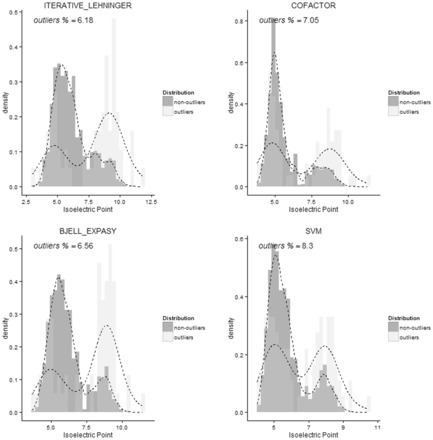
Distribution of outliers and non-outliers populations of the PIPDB portion evaluated. An outliers is defined if: Abs(p*I*_experimental_ – p*I*_theoretical_) ≥ SD (p*I*_theoretical_)

A potential use of p*I* prediction algorithms is the possibility to detect outliers from experimental data and detect possible incorrect assignment at the protein and peptide level (Perez-Riverol *et al.*, 2011). The number of outliers also can be use as quality assessment metric of the separation technique (Ramos *et al.*, 2011). If the algorithm is more accurate it tends to predict more outliers and possible false-positive identifications assignments. Figure 4 shows the distribution of outliers and non-outliers, of four of the p*I* algorithms under study. The SVM-based algorithm proposed by Perez-Riverol and co-workers in 2012 predicted the percentage of outliers, especially in the neutral and basic regions, where the method out-performs the other algorithms.

### 3.2 Isoelectric point estimation of peptide sequences

**Fig. 5.**
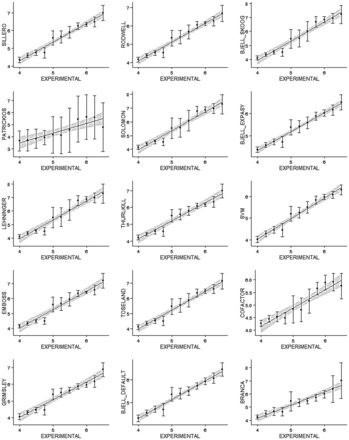
Experimental versus theoretical isoelectric point for 11 different peptide fractions of an OFF-GEL electrophoresis experiment (Heller dataset)

Results seen for the peptide dataset are markedly different: a high correlation was observed for most methods (Table 1). Although the top seven methods all show the same correlation *R*^2^^ ^= 0.96, the best result is the SVM method (Perez-Riverol *et al.*, 2012) which has the lowest RMSD (0.21). Figure 5 shows the average p*I* and standard deviation from IEF fractions from the peptide dataset analyzed. The largest standard deviation was found in the 5–7 p*I* range. The best correlation is always observed in the acid fractions (Fig. 4). In this region, most of the methods generated a good estimate for the peptides, with a p*I* between 4.0 and 5.0*,* where the lowest RMSD was obtained. Similar to the results obtained on PIP-DB analysis, the Iterative method used with the Patrickios p*K*_a_ set fails fully in the complete dataset.

The Bjellquivst algorithm shows a higher number of outliers (black dots) in the basic fractions compare with the SVM algorithm (Supplementary Information S2, Fig. 2). Outlier (peptide with deviant p*I*) detection is highly dependent on both the accuracy of the p*I* estimation and the quality of the electrophoretic experiment (Ramos *et al.*, 2011). Predicted p*I*s can be applied to remove less likely identified peptides and to curate the final protein identification result lists in a shotgun proteomics experiment (Perez-Riverol *et al.*, 2011). Importantly an outlier may not necessarily imply an estimation error, but may indicate the presence of one or more posttranslational modifications (Lengqvist *et al.*, 2011).

Interestingly, no major changes in the correlation values are observed when different p*K*_a_ sets are used with the Bjellqvist method, 0.95–0.96; this suggests that most of the p*K*_a_s values published after Bjellqvist (Bjellqvist *et al.*, 1993) only perform better in certain analytical settings (Table 1). A similar trend is observed for the Iterative method; this again suggests that none of the sets of p*K*_a_ values is optimal or is necessarily superior to any other, and in most cases different p*K*_a_ values should be used for different calculations. The cofactor method (Fig. 4) performs more poorly than most of the algorithms as it was designed to study acid fractions only (Cargile *et al.*, 2008). The recent Branca algorithm also exhibited a poor performance on this dataset (*R*^2^^ ^= 0.85, Table 1). It was also designed and trained to study peptides in acid fractions by adding corrections to the original p*K*_a_ sets proposed by Bjellquivst. For this reason, in basic ranges the algorithm has a low correlation coefficient and a high RMSD value (Fig. 4).

In contrast, other methods such as SVM, Bjellqvist and Iterative (with certain p*K*_a_ sets) show better behavior throughout the fractions analyzed. The confidence intervals indicating the ability to predict near to an ideal performance (p*I*_experimental_ versus p*I*_predicted_) are closest for these last algorithms.

### 3.3 Isoelectric point estimation of modified peptides

**Fig. 6.**
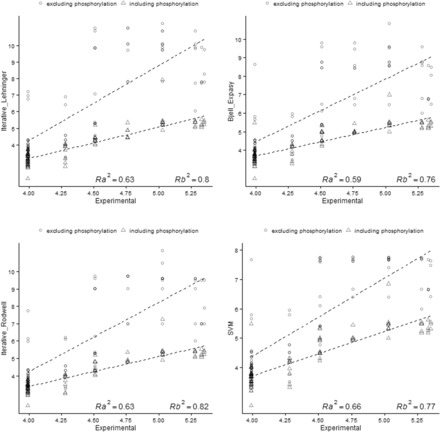
Correlation between predicted p*I* versus experimental p*I.* The plots show the correlation obtained if Phosphorylation is exclude (○) or include (Δ) in the p*I* calculation. *R*_a_ and *R*_b_ denote the correlation coefficients excluding and including the modification in the estimation respectively. The p*K*_a_ and p*K*_b_ values of 1.2 and 6.5 for phosphorylation S and T were used to consider the phosphorylation effect in the p*I* estimation

**Fig. 7.**
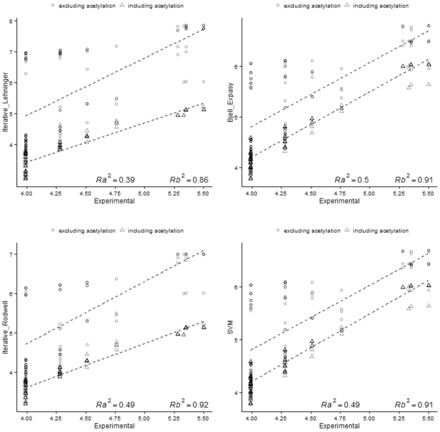
Correlation between predicted p*I* versus experimental p*I.* The plots show the correlation obtained if N-terminal acetylation is exclude (○) or include (Δ) in the p*I* calculation. *R*_a_ and *R*_b_ denote the correlation coefficients excluding and including the modification in the estimation respectively. The p*I* of the N-terminal acetylated peptides was calculated by omitting the p*K* values of the N-terminal residue in the peptide sequence

Post-translational and experimentally induced peptide modifications can shift peptide p*I* compared to the values estimated for the unmodified sequence in two ways: (i) by introducing charged groups or (ii) by neutralizing charged groups (Ramos *et al.*, 2011). The results presented in Section 3.2, show for most of the algorithms, a good correlation between the predicted and experimental p*I* values of peptides on a ‘non-modified’ dataset. However, an extended analysis taking into account post-translational modifications such as phosphorylation and N-terminal acetylation will provide a more accurate representation of a real electrophoretic experiment. Figures 6 and 7 show the experimental p*I* values versus the predicted phosphopeptide and acetylated peptide p*I* and also the non-modified variants for several p*I* algorithms, with a previously reported dataset (Gauci *et al.*, 2008). The best correlation was obtained when the modification was considered during the estimation of the theoretical p*I*, increasing the correlation from 0.4 to 0.9 for most of the algorithms (Supplementary Information S3), demonstrating the dramatic effect of post-translational modifications on p*I* estimation.

When the N-terminus of a peptide is acetylated, a positive charge is lost, decreasing the overall charge of the peptide (Gauci *et al.*, 2008; Lengqvist *et al.*, 2011). Correspondingly, phosphorylation affects the charge of a peptide by adding a negatively charged group (Halligan, 2009; Ramos *et al.*, 2011). Figure 7 shows that the impact of acetylation is more predominant to phosphorylation, making results poorer if acetylation is not take into account. If those PTMs are not considered during predictions and *in silico* studies, the final results can be completely different to the real experiment, especially, the acetylation due the distribution of Lysine and N-terminally in tryptic proteomes (Perez-Riverol *et al.*, 2011).

## 4 Discussion

Our benchmarking comparison constitutes a strong blind test, since no method is in any way optimized for this particular data set and all the proteins and corresponding isoelectric points were collected from different sources and correspond to distinct analytical settings. The present study demonstrates that the algorithms from Bjellqvist *et al.* and Perez-Riverol *et al.* represent the most accurate algorithms overall for computing protein isoelectric points. The results also demonstrate that when other p*K*_a_ sets are employed no significant differences were seen for the Bjellqvist *et al.* approach, and most of the small differences observed can be related to specific analytical conditions specific to the experiment. The poor performance of all evaluated algorithms for the single value protein dataset is alarming. There are several possible explanations for this behaviour. The dataset was evaluated using a variety of methods over many decades. Thus the intrinsic variation will arise both from true biological variation (the presence of unknown processed or truncated proteins or proteins with charged post-translational modifications, etc.) and other errors introduced by a gallimaufry of different experimental protocols, each with distinct and incommensurable calibration. The low correlation between experimental and predicted values throws serious doubt on the veracity of many theoretical studies of isoelectric point distributions in whole proteomes, such as the studies by Wu *et al*. (2006) and Carugo (2007), and any arguments made on that basis. An alternative exegesis posits that PIP-DB must contain a large number of annotation errors, as introduced during database construction, which seriously contaminates the result. This is clearly possible, if highly unlikely.

In IEF gels or SDS-PAGE experiments, it is common to find the same polypeptide instance through multiple experimental fractions, due to diffusion phenomena, or from uneven cutting during band excision across of the gel. In addition, the peptides or proteins could have precipitated out of solution during migration from the well to the appropriate pH in the gel.

Possible aggregation and degradation could contribute to enhance inappropriate focalization, making it difficult to correctly interpret the results obtained. It has also been shown that not only the amino acid composition but also its subsequent modification can influence the accurate estimation of the isoelectric point, e.g. common modifications such as phosphorylation and acetylation which might lead to the shielding of surface charges (as previously described in Section 3.3). PIP-DB illustrates this complex scenario as shown in the Table 3. It contains multiple proteins that may be ‘detectable’ in both acid and basic region, showing a wide focalization zone. Most p*I* prediction methods do not take into account such ‘artifacts’ and will thus fail to make accurate estimates.

**Table 3:** Proteins with a wide focalization zone in PIP-DB

Protein	Occurrences	Focalization zone size*
gi|15924609	3	3.5
sp|P09616	5	3.5
sp|Q02161	3	3.5
sp|Q3T0P6	3	3.65
sp|P12019	3	3.7
tr|Q9N102	4	3.79
sp|P00750	2	3.8
sp|P23141	3	3.8
sp|Q8WVB3	28	3.8
tr|Q9XDT2	2	3.9
sp|P31211	6	4.0
sp|P00558	3	4.05
tr|Q6LBH1	30	4.08
sp|O00142	3	4.1
gi|2506195	2	4.2
sp|P24735	6	4.3
tr|Q9AJM4	2	4.3
sp|P06746	4	4.35
sp|P55056	2	4.39
sp|P01139	3	4.8
sp|P54819	10	4.8
sp|P00480	5	4.85
sp|P13233	23	4.9
sp|P14518	5	4.9
tr|E5GC51	8	6.4

* Defined by the difference between the maximal and minimal p*I* experimental value found for the protein in PIP-DB.

These results also highlight the need for a complete and customizable tool that can provide all available algorithms and p*K*_a_ sets for isoelectric point analysis. The machine-learning algorithms, especially the SVM-based algorithm, showed a superior performance when studying peptide mixtures. In general, learning-based p*I* prediction methods (such as Cofactor, SVM and Branca) require a large training dataset and their resulting performance will strongly depend of the quality of that data. Even though Cofactor and Branca algorithms are based on learning approaches, it is not possible to retrain these algorithms with different datasets. In this sense, the SVM approach shows more ‘flexibility’ in p*I* computation, and this feature can improve prediction accuracy. In contrast with Iterative methods, machine-learning algorithms have the advantage of being able to add new features to improve prediction. Considering the amount of p*K*_a_ sets reported to date, use of the Iterative approach provides a good opportunity to find some variant that fits well with particular experimental conditions. In the near future, new algorithms and bioinformatics tools should be able to provide a way of choosing different p*K*_a_ sets and thus obtain more accurate prediction for a given analytical setting. The SVM and Cofactor methods should only be used where a high number of sequences are studied and can thus be used to train the algorithms. We observed notable fluctuations in p*I* predictions for Iterative methods on a small dataset, showing this method to be sensitive to small changes in the amino acid p*K*_a_ values used. Moreover, the algorithm fails when certain p*K*_a_ values are missing. We envisage that more algorithms based on machine learning, including new additional features, should be explored allowing the development of fast, accurate and reliable p*I* calculation algorithms for use in future protein and peptide proteomic analysis.

## Funding

Y.P-R. is supported by the BBSRC ‘PROCESS’ grant [BB/K01997X/1].

*Conflict of Interest*: none declared.

## Supplementary Material

Supplementary Data
