# Supplementary material for: Accurate estimation of isoelectric point of protein and peptide based on amino acid sequences
Source: Bioinformatics. 2015 Nov 14;32(6):821–7. doi: 10.1093/bioinformatics/btv674 (PMC5939969; doi:10.1093/bioinformatics/btv674)
Supplement: Supplementary Data [file btv674_supplementary_data.zip › Supp_Information_S2.docx]

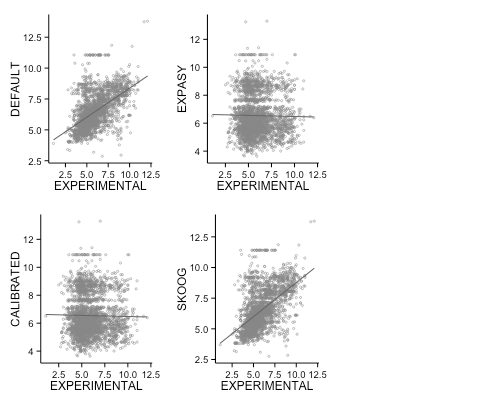


Figure 1: Correlation between theoretical and experimental isoelectric points using the Bjellquivst method using the complete PIP-DB.


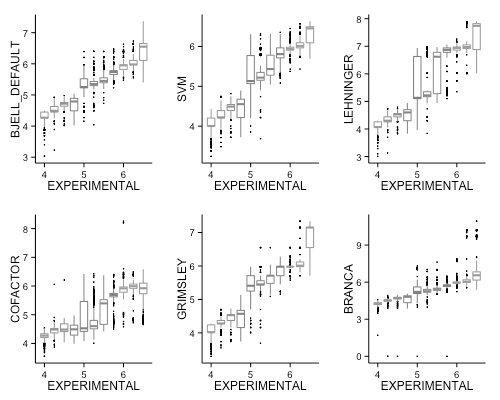


Figure 2: Experimental vs. theoretical isoelectric point for 11 different peptide fractions of an OFF-GEL electrophoresis experiment (peptide dataset). The black dots represent the outliers for each OFF-GEL fraction.
