# Supplementary material for: Accurate estimation of isoelectric point of protein and peptide based on amino acid sequences
Source: Bioinformatics. 2015 Nov 14;32(6):821–7. doi: 10.1093/bioinformatics/btv674 (PMC5939969; doi:10.1093/bioinformatics/btv674)
Supplement: Supplementary Data [file btv674_supplementary_data.zip › Supp_Information_S1.docx]

**Theoretical and practical key notes about isoelectric point prediction algorithms.**

1. **Bjellqvist’s *pI* algorithm**

The more extended algorithm used for polypeptides isoelectric point prediction was proposed firstly by Bjellqvist and coworker ([Bjellqvist, Hughes et al. 1993](#_ENREF_1)). It is actually, the base of the **Compute pI tool** in the computational proteomics suite Expasy (http://web.expasy.org/compute_pi/). Like subsequent algorithms for the polypeptides isoelectric point prediction, the Bjellqvist algorithm is based on the Henderson-Hasselbalch equation (1) which express the relationship between pH and the ionization constant (*pK*) of carboxylic acid groups (*A*) or amino groups (*B*).

$pH=pK+\log\left( \frac{\left[ A^{-} \right]}{\left[ AH \right]} \right)$ or $pH=pK+log\left( \frac{\left[ B \right]}{\left[ {BH}^{+} \right]} \right)$ (1)

The equations (2), (3) and (4) listed below, complete this theoretical framework and support the main core of the currently *pI* algorithms estimation.

$\alpha_{A}= \frac{\left[ A^{-} \right]}{\left[ A^{-} \right]+ \left[ AH \right]}$ or $\alpha_{A}= \frac{\left[ {BH}^{+} \right]}{\left[ {BH}^{+} \right]+ \left[ B \right]}$ (2)

Where 𝛼 is defined as the charged fraction for each ionizable group in a polypeptide.

$\alpha_{A}= \frac{-1}{{10}^{pK-pH}+ 1}$ or $\alpha_{B}= \frac{-1}{{10}^{pH-pK}+ 1}$ (3)

Equation (3) is derived from equations (1) and (2) and is used to compute 𝛼 for each ionizable group at a given pH.

$Q_{n}= \sum_{i=1}^{A} \frac{-1}{{10}^{pK-pH}+ 1}+\sum_{i=1}^{B} \frac{-1}{{10}^{pH-pK}+ 1}$ (4)

The *pI* of a polypeptide is the pH at which the net charge of the polypeptide equals 0 and can be calculated using equation (4), which gives the net charge (*Q_n_*) of a polypeptide at a given pH.

1. **Iterative *pI* algorithm**

In general, an Iterative algorithm to predict the isoelectric point value works following three major steps. Firstly, it count the numbers of copies of the amino acids which play a role in determining pI and to propose pH values. Second, it determines the expected charge on the polypeptide for a particular pH value. And finally, it determines the expected proportion of charged and uncharged side chains for a particular amino acid, which are assessed from the supplied *pK* values. As show the table 1, there are several *pK* sets defined that could be used according to experimental setting evaluated.

**Table 1.** pK sets reported in the literature. It are used in Iterative algorithm for pI estimation.

|  | pk values | | | | | | | | |
| --- | --- | --- | --- | --- | --- | --- | --- | --- | --- |
|  | C-Term | N-Term | D | E | K | R | H | C | Y |
| Rodwell ([Rodwell 1982](#_ENREF_9)) | 3.1 | 8.0 | 3.86 | 4.25 | 11.5 | 11.5 | 6.0 | 8.33 | 10.07 |
| Sillero ([Sillero and Maldonado 2006](#_ENREF_10)) | 3.2 | 8.2 | 4.0 | 4.5 | 10.4 | 12.0 | 6.4 | 9.0 | 10.0 |
| Solomon ([Solomons and Fryhle](#_ENREF_11)) | 2.4 | 9.6 | 3.9 | 4.3 | 10.5 | 12.5 | 6.0 | 8.3 | 10.1 |
| Patrickios ([Patrickios and Yamasaki 1995](#_ENREF_7)) | 2.4 | 9.6 | 4.2 | 4.2 | 11.2 | 11.2 | - | - | - |
| Lehninger ([Nelson, Lehninger et al. 2008](#_ENREF_6)) | 2.34 | 9.69 | 3.86 | 4.25 | 10.5 | 12.4 | 6.0 | 8.33 | 10.0 |
| Grimsley ([Grimsley, Scholtz et al. 2009](#_ENREF_4)) | 3.3 | 7.7 | 3.5 | 4.2 | 10.5 | 12.0 | 6.6 | 6.8 | 10.3 |
| Toseland ([Toseland, McSparron et al. 2006](#_ENREF_14)) | 3.19 | 8.71 | 3.6 | 4.29 | 10.45 | 12.0 | 6.33 | 6.87 | 9.61 |
| Thurlkill ([Thurlkill, Grimsley et al. 2006](#_ENREF_13)) | 3.67 | 8.0 | 3.67 | 4.25 | 10.4 | 12.0 | 6.54 | 8.55 | 9.84 |

A popular approach to compute the isoelectric point following this way, is implemented in the **DTASelect** tool ([Tabb, McDonald et al. 2002](#_ENREF_12)) and summarized in the figure 1. First, it calculates the charge of a protein at pH 7. Then next proposed pH moves 3.5 (half of 7) higher or lower depend on the charge. In the next jump, the proposal moves 1.75 (half of 3.5) in the appropriate direction. These jumps continue until the charge is appropriately close to zero. The search space is halved with each jump, resulting in an efficient search for the isoelectric point.

From this procedure derivate some limitations on the accuracy prediction of the isoelectric point. First, by adding together the charges in the above described way, the algorithm assumes that each group’s charge is independent of all others; if the protein contains an arginine side chain and an aspartic acid side chain, for example, these two groups are assumed to take on a charge irrespective of their locations in the protein sequence. If a basic residue is adjacent to an acidic residue, each probably does change the other’s ability to take on a charge, but this effect is ignored in this algorithm.

**
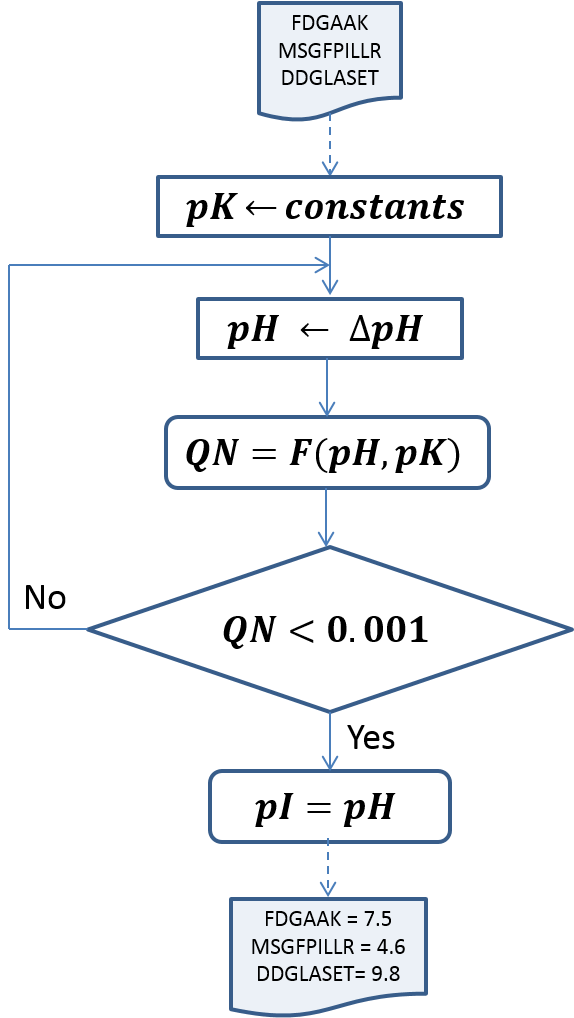
**

**Figure 1.** Simplified flow**-**diagram of Iterative algorithm to isoelectric point prediction.

1. **Cargile’s *pI* algorithm**

The isoelectric point prediction algorithm implemented by Cargile and coworkers ([Cargile, Sevinsky et al. 2008](#_ENREF_3)), was the first proposed approach in “modelling” the effect of interaction between charged neighboring amino acids; showing that the electrostatic interactions between adjacent amino acid residues in polypetides play a major role in determining the accuracy of the *pI* calculation.

The Cargile’s algorithm (**Cofactor**) accounts the effect of adjacent amino acids ± 3 residues away from a charged aspartic (Asp) or glutamic acid (Glu), the effects on free C-terminus, as well as a correction term applied to the corresponding *pK* values. For developing this algorithm, the authors be based on a machine learning method (genetic optimization method) with a 5,000-peptide training set. The accuracy of the new *pI* values obtained was close to the error derived from the manufacturer of the IPG strips. However, it is important nothing that the algorithm and the adjusted *pK* values were optimized only in the acid *pH* range (from 3.5 to 4.5), which is a weak when it is used in overall pH range.

1. **Branca’s *pI* algorithm**

Similar to Cargile *pI* algorithm, the Branca´s approach ([Branca, Orre et al. 2014](#_ENREF_2)) improves the isoelectric point estimation by to introduce two new optimizations on the *pK* scale. First, it introduced *pK* value correction by considering the influence of neighboring ionizable groups up to six residues away through amino acid sequence. And second, the algorithm also introduces the use of a statistical correction factor that depends on the number and type (Asp or Glu) of carboxylic acid side chains existing in the peptide. Both optimizations are applied on original *pK* values proposed by Bjellqvist and the *pK* value corrected are used for computing the pI value. The optimization process was done using high-resolution isoelectric focusing (HiRIEF) data, and the final *pK* constants used to predict the isoelectric point, was done by changing these constants in small discrete steps and verifying the impact on the prediction accuracy on large training set.

An additional feature of this algorithm (available like **PredpI tool**), is the possibility of to estimate the *pI* using three different *pK* sets. For unmodified peptide, the authors put forward the *pKconstants-plain* set, in the same way, both iTRAQ- as TMT-modified peptides *pI* values can be predicted using *pKconstants-iTRAQ* and *pKconstansts-TMT* in that order.

1. **SVM *pI* algorithm**

Perez-Riverol and coworkers ([Perez-Riverol, Audain et al. 2012](#_ENREF_8)) developed a machine learning-based approach to estimate the peptide or protein isoelectric point. The model uses Support Vector Machines (SVM) as predictor, and takes into account both an experimental amino acid descriptor from the AAIndex database ([Kawashima, Pokarowski et al. 2008](#_ENREF_5)) and the isoelectric points predicted by the Bjellqvist model.

The **figure 2** summarize the flow of the algorithm follow until getting a final SVM model trained. In brief, from a dataset containing polypeptides with their experimental *pI* value associated, it is randomized and split into two subset: training dataset and test dataset. In addition, the test stage include a step of cross-linked validation to get the “best classifier”. The selection criteria used to choose the best classifier is the lowest root-mean-squared-error (RMSE) reached in this step, and *i* variable define the folds that the process is repeated. Taking into account the results of previous runs on different dataset, choosing a value to *i = 10,* seems optimal to guarantee a fast convergence and good outcome of the algorithm. With the new model is possible to predict the *pI* for new instances (peptides or proteins).

**
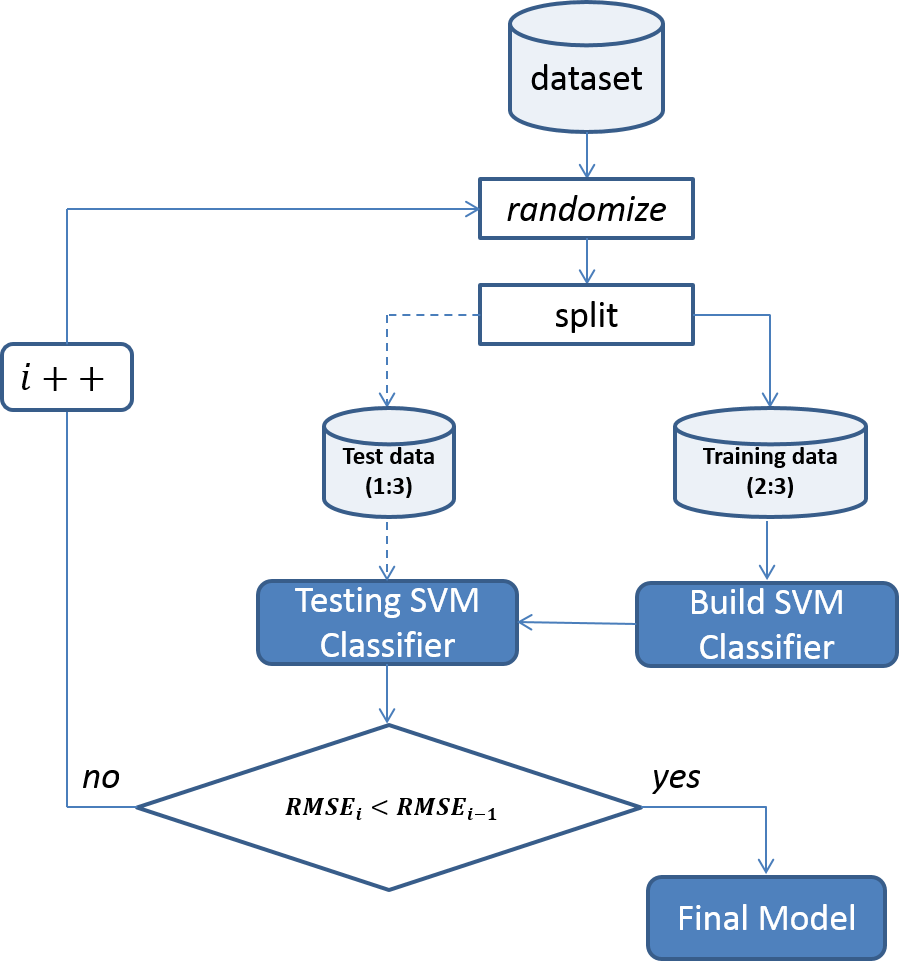
**

**Figure 2.** Schematic representation of SVM-based algorithm for isoelectric point estimation.

1. **References**

**Bjellqvist, B., et al. (1993). "The focusing positions of polypeptides in immobilized pH gradients can be predicted from their amino acid sequences." Electrophoresis 14(10): 1023-1031.**

**Branca, R. M. M., et al. (2014). "HiRIEF LC-MS enables deep proteome coverage and unbiased proteogenomics." Nat Meth 11(1): 59-62.**

**Cargile, B. J., et al. (2008). "Calculation of the isoelectric point of tryptic peptides in the pH 3.5-4.5 range based on adjacent amino acid effects." Electrophoresis 29(13): 2768-2778.**

**Grimsley, G. R., et al. (2009). "A summary of the measured pK values of the ionizable groups in folded proteins." Protein Sci 18(1): 247-251.**

**Kawashima, S., et al. (2008). "AAindex: amino acid index database, progress report 2008." Nucleic Acids Res 36(Database issue): D202-205.**

**Nelson, D. L., et al. (2008). Lehninger principles of biochemistry, Macmillan.**

**Patrickios, C. S. and E. N. Yamasaki (1995). "Polypeptide amino acid composition and isoelectric point. II. Comparison between experiment and theory." Anal Biochem 231(1): 82-91.**

**Perez-Riverol, Y., et al. (2012). "Isoelectric point optimization using peptide descriptors and support vector machines." J Proteomics 75(7): 2269-2274.**

**Rodwell, J. D. (1982). "Heterogeneity of component bands in isoelectric focusing patterns." Anal Biochem 119(2): 440-449.**

**Sillero, A. and A. Maldonado (2006). "Isoelectric point determination of proteins and other macromolecules: oscillating method." Comput Biol Med 36(2): 157-166.**

**Solomons, T. G. and C. Fryhle Organic chemistry, 2000, John Wiley & Sons, Inc., USA.**

**Tabb, D. L., et al. (2002). "DTASelect and Contrast: tools for assembling and comparing protein identifications from shotgun proteomics." J Proteome Res 1(1): 21-26.**

**Thurlkill, R. L., et al. (2006). "pK values of the ionizable groups of proteins." Protein Sci 15(5): 1214-1218.**

**Toseland, C. P., et al. (2006). "PPD v1.0--an integrated, web-accessible database of experimentally determined protein pKa values." Nucleic Acids Res 34(Database issue): D199-203.**
