# Supplementary material for: Accurate estimation of isoelectric point of protein and peptide based on amino acid sequences
Source: Bioinformatics. 2015 Nov 14;32(6):821–7. doi: 10.1093/bioinformatics/btv674 (PMC5939969; doi:10.1093/bioinformatics/btv674)
Supplement: Supplementary Data [file btv674_supplementary_data.zip › Supp_Information_S3.docx]

1. **N-terminal acetylation effect on Isoelectric point (*pI*) prediction.**


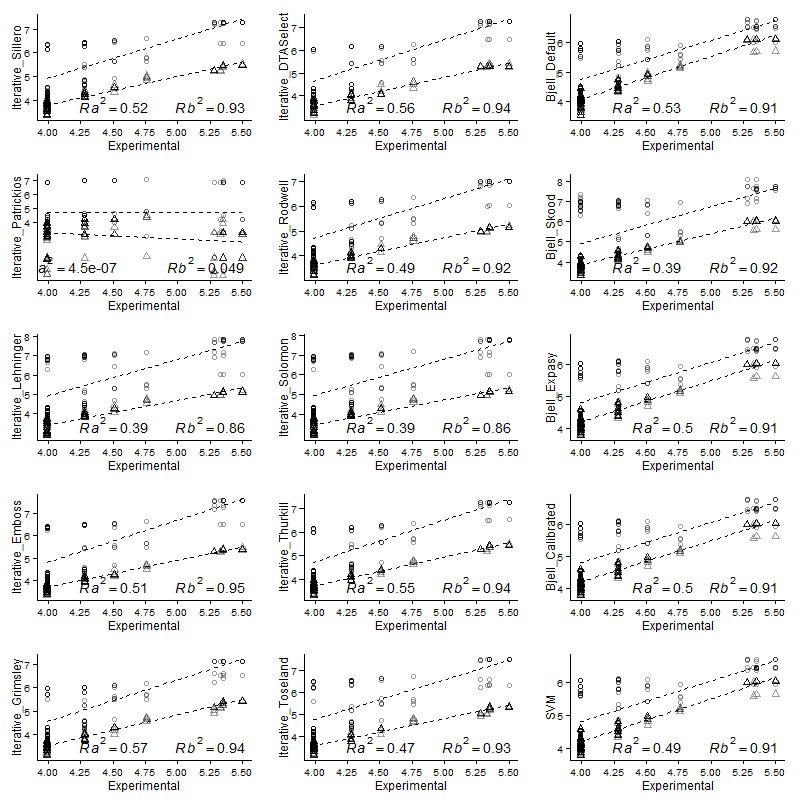


**Figure 1.** Correlation between predicted pI versus experimental pI. The plots show the correlation obtained if **N-terminal acetylation** is exclude (○) or include (∆) in the pI calculation. R_a_ and R_b_ denote the correlation coefficients excluding and including the modification in the estimation respectively. The pI of the N-terminal acetylated peptides was calculated by omitting the pK values of the N-terminal residue in the peptide sequence.

1. **Phosphorylation effect on Isoelectric point (*pI*) prediction.**


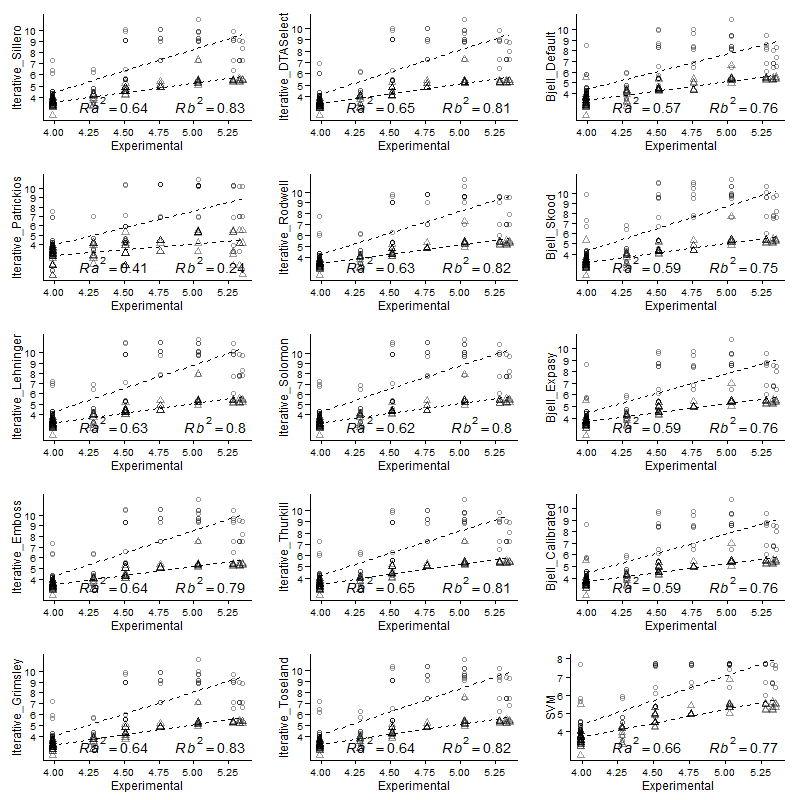


**Figure 2.** Correlation between predicted pI versus experimental pI. The plots show the correlation obtained if **Phosphorylation** is exclude (○) or include (∆) in the pI calculation. R_a_ and R_b_ denote the correlation coefficients excluding and including the modification in the estimation respectively. The pKa and pKb values of 1.2 and 6.5 for phospho S and T were used to consider the phosphorylation effect in the pI estimation.
